# Error Analysis of Terrestrial Laser Scanning Data by Means of Spherical Statistics and 3D Graphs

**DOI:** 10.3390/s101110128

**Published:** 2010-11-10

**Authors:** Aurora Cuartero, Julia Armesto, Pablo G. Rodríguez, Pedro Arias

**Affiliations:** 1 Polytechnic School, University of Extremadura, 10071 Cáceres, Spain; E-Mail: pablogr@unex.es (P.G.-R.); 2 Mining Engineering School, University of Vigo, 36310 Vigo, Spain; E-Mails: julia@uvigo.es (J.A.); parias@uvigo.es (P.A.)

**Keywords:** accuracy, terrestrial laser scanning (TLS), angular analysis, spherical statistics

## Abstract

This paper presents a complete analysis of the positional errors of terrestrial laser scanning (TLS) data based on spherical statistics and 3D graphs. Spherical statistics are preferred because of the 3D vectorial nature of the spatial error. Error vectors have three metric elements (one module and two angles) that were analyzed by spherical statistics. A study case has been presented and discussed in detail. Errors were calculating using 53 check points (CP) and CP coordinates were measured by a digitizer with submillimetre accuracy. The positional accuracy was analyzed by both the conventional method (modular errors analysis) and the proposed method (angular errors analysis) by 3D graphics and numerical spherical statistics. Two packages in R programming language were performed to obtain graphics automatically. The results indicated that the proposed method is advantageous as it offers a more complete analysis of the positional accuracy, such as angular error component, uniformity of the vector distribution, error isotropy, and error, in addition the modular error component by linear statistics.

## 1. Introduction

In the last decade, terrestrial laser scanning (TLS) systems have appeared on the market and found a firm place in geodetic metrology. When TLS laser scanners were introduced on the market, their performances were rather poor, having in general a measurement uncertainty in the range of centimeters. However, with the progressive improvement of technology and the consequent increase in the measurement precision, the potential range of purposes has been widened from some meters to hundreds of meters in forensics [1], forestry [2], environment [3,4] geology [5], structure analysis [6,7], ship building [8] and archaeological applications [9,10]. A complete overview of the TLS technology and processing methods, as well as applications, is presented in Volsseman and Maas [11]. Further Lemmens [12] shows an updated description of different commercial instruments and their technical characteristics. If any metric data are obtained from the scanned data, the errors can be known. The need for calibration has been widely stated [13]. However, for active sensors, standards for error evaluation have not been established yet. With the publication of ISO standard 17123 part 8 (GNSS field measurement systems in Real Time Kinematic –RTK–) in September 2007, TLS are the only remaining geodetical measuring systems without standardised field test procedures. In accordance with the chair of ISO TC172/SC6 and with the support of Leica Geosystems AG Heerbrugg, Switzerland, basic ideas for simplified and full field test procedures for TLS have been worked out in a diploma thesis at the University of Applied Sciences Northwestern Switzerland [14]. Basically, the computed (experimental) standard deviations are compared on the basis of statistical tests. The most important results from the thesis are summarised by Gottwald [15]. The use of these proposals is under evaluation by the ISO Technical Committee (IS0 TC172/SC6).

As a result of the absence of standards, the accuracy specifications given by laser scanner producers in their publications and pamphlets are not comparable [16]. Experience shows that sometimes these should not be trusted. The instruments that are built in a small series vary from instrument to instrument and depend on the individual calibration and the care that has been taken in handling the instrument [17]. Furthermore, the terms error, accuracy, and precision are sometimes misused.

The first suggestion for system calibrations, system tests and accuracy checks for TLS correspond to Lichti [18,19]. Most of the published investigations are based on field or laboratory tests [20,16]. Some researchers had already published methods and results concerning accuracy tests with laser scanners [19,21,22].

Reshetyuk [23] estimated the position of the target centre from a number of points, then performed self-calibration of different scanners, and the rigid body transformation parameters between the scanner and external coordinate systems for all of the scans were estimated, as well as the calibration parameters, in a parametric least squares (LS) adjustment and the coordinate “3D residuals”.

The “technical” parameters representing the mechanical-optical stability such the geometry of the axes, eccentricity, and the addition constant were obtained for certain instruments [24]. For the accuracy of the distance measurement, truth and measured distance were compared, obtaining standard deviations.

Mechelke *et al.* [25] present an investigation into the accuracy behaviour through derived distances from point clouds of a 3D test field for accuracy evaluation of 3D laser scanning systems, accuracy tests of distance measurements in comparison to reference, accuracy tests of inclination compensation, the influence of the laser beams angle of incidence on 3D accuracy, investigations into scanning noise and investigations into the influence of object colour in distance measurements.

Kersten *et al.* [20] obtained the average and maximum deviation to the sphere and target centres (prior alignment) as well as the comparison of the distances determined in all combinations between reference points. Furthermore, the trunnion error and influence of the colour and material of the scanned surface were evaluated.

Lichti [26] presented the full mathematical model for a point-based photogrammetric approach to FARO LS880HE TLS self-calibration. Schneider [27] presents the calibration and analysis of a terrestrial laser scanner Riegl LMS-Z420i, showing the precision improvement of the adjusted observations as a result of a stepwise addition of calibration parameters.

The International Organization for Standardization (ISO) was formed in 1947 as a non-governmental federation of standardization bodies from over 60 countries. The ISO is headquartered in Geneva, Switzerland. The Unites States is represented by the ANSI.

In 1984, ISO published the 1st edition of the “International vocabulary metrology _Basic and general concepts and associated terms (VIM)” [28]. International standards of ISO 5725-1 [29] present general principles and definitions about metrological concepts.

It considers appropriate to review some terms:

▪ Precision: degree of closeness between independent measurement results obtained *in particular* established conditions and depends on random factors only. The measure of precision is usually calculated as standard (root-mean-square) deviation of results of measurements performed in definite conditions. Precision depends only on the distribution of random errors and does not relate to the true value or the specified value. The measure of precision is usually expressed in terms of imprecision and computed as a standard deviation of the test results.
▪ Accuracy: The closeness of agreement between a test result and the accepted reference value. The term accuracy, when applied to a set of test results, involves a combination of random components and a common systematic error or bias component.
▪ Uncertainty, parameter associated with the result of a measurement, that characterizes the dispersion of the values that could reasonably be attributed to the measurand. The parameter may be, for example, a standard deviation, or the half-width of an interval having a stated level of confidence.

The most common descriptor in geosciences is the root mean square error (RMSE). The frequently used mean error (ME) and error standard deviation (S) are also given in accuracy tests for a more complete statistical description of errors. However, none of these descriptive statistics (RMSE, ME, S) reports more than a global summary statistic based on comparison with a limited sample of points and only from the perspective of analysing the modulus (vertical and horizontal are not considered) of such errors. The two angles of error can be found through statistical analysis of spherical data. This approach to error evaluation has been used in the earth sciences [30], geology [31], biology [32], meteorology [33], palaeomagnetism [34], electronics [35] and biomechanics [36]. The statistical analysis of spherical data started with R.A. Fisher [37], who developed a distribution for angular errors on a sphere. N.I. Fisher [38] investigated various properties of the spherical median and discussed equivalents for the sign test. Later, the book [39] was devoted purely to the analysis of spherical data.

While several authors have contributed to providing accuracy evaluations of 3D laser scanning systems, 3D statistic analysis has been with available scanner data. In brief, the aim of this work is to present a novel proposal to analyse the positional accuracy in TLS data with a more complete analysis than currently available. Our proposal is characterised by some issues: the use of check points (CP) acquired by a technology with more accuracy (Proliner) and error analysis by means of spherical statistics.

## 2. Methodological Proposal for Error Analysis

In this proposal, an alternative way to analyse the error by means of spherical statistics is presented. The error of a control point “i” is defined as the value e_i_ = c_i_ − c_j_, where c_i_ is the coordinate point measured and c_j_ the “real” or “true” coordinate, estimated by more precise methods.

**Figure 1.**
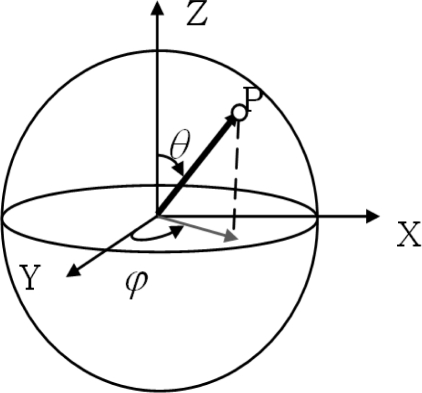
TLS Intrinsic Coordinates System.

The deviation between the true position (true data) and the corresponding point with TLS data is estimated as a vector. Each vector is defined by means of its modulus and two angles (colatitude and longitude—inclination and azimuthal), which allows us to analyse the errors in a 3D space or spherical coordinates, like the type of measured TLS data. Spherical coordinates, also called spherical polar coordinates, are a system of curvilinear coordinates that are natural for describing positions on a sphere (Figure 1).

The pairwise comparison of measured and reference coordinates allows the calculation of the ME, S, RMSE or similar statistics. RMSE is the square root of the average of the set of squared differences between the dataset coordinate values and coordinate values from an independent source of higher accuracy for identical points.

The statistical procedure proposed includes the basic statistic calculations (for modular and angular data) and the main tests for spherical distributions. The error analysis proposed in this paper consists of several parts. In the first part, the modular error component was analysed by linear statistics, similar to the conventional method. In the second part, the angular error components were analysed as well. In the third part, the most innovative part of the analysis, the graphical analysis was developed by 2D and 3D graphics with two packages of the R programming language. In the last part, a study of the uniformity and normality of the distribution error data was done to complete the data analysis.

### 2.1. Analysis Statistic of Modular Error

The error is a vector with three Cartesian components, one for each axis X, Y and Z, and denoted Δ*x*, Δ*y* and Δ*z*, respectively. The modular error (Δ*m*) is the magnitude equivalent to the square root of the sum of the squares of the previous terms:

(1) $$\Delta m=\sqrt{\Delta x^{2}+\Delta y^{2}+\Delta z^{2}}$$

The basic statistics of the modulus (no angles are considered) are the sample mean, minimum and maximum values, standard deviation and root mean square error (RMSE).

▪ The sample mean (*μ*) is calculated by taking the sum of all the data values and dividing by the total number of data values. The sample mean is a measure of location, commonly called the average:
  (2) $$\mu =\frac{1}{n}\sum_{i=1}^{n}e_{i}$$
▪ The range of errors is the difference between the largest (maximum) and the smallest (minimum) calculated error. It is a measure of the spread or the dispersion of the error observations.

There are several measures of dispersion, the most common being the standard deviation. These measures indicate to what degree the individual observations of a data set are dispersed or ‘spread out’ around their mean.

▪ The standard deviation (S or SD) is calculated by taking the square root of the variance. The sample variance is the sum of the squared deviations from their average divided by one less than the number of observations in the data set. It is a measure of the spread or dispersion of a set of data. In the measurements, high precision is associated with low dispersion:
  (3) $$s=\sqrt{\frac{1}{n}{\left(e_{i}-\mu \right)}^{2}}$$
▪ The root mean square error (RMSE), or standard error, is the square root of the average of the set of squared differences between dataset coordinate values and coordinate values from an independent source of higher accuracy for identical points. RMSE is a good measure of accuracy:
  (4) $$\mathrm{RMSE}=\sqrt{\frac{1}{n}\sum_{i=1}^{n}e_{i}{}^{2}}$$

The RMSE is an expression equivalent to the SD in the absence of bias (*i.e*., if = 0). It is important to calculate both magnitudes, SD and RMSE, because the first refers to the precision and the second to the accuracy.

### 2.2. Analysis Statistic of Angular Errors

Both a magnitude and a direction must be specified for a vector quantity, in contrast to a scalar quantity, which can be quantified with just a number. In the same way that a vector has three Cartesian components, it can also be decomposed into polar components: modular distance, vertical angle and horizontal angle. The modular component was analysed in the previous step (Section 2.1).

There are different conventions for considering the angles. In this work, the following convention is proposed because it is the most appropriate to TLS and similar to geographical nomenclature (Figure 1):

▪ The vertical angle (*θ*) is the angle measured clockwise from the positive z-axis to a point with 0 ≤ *θ* ≤ *π*
▪ The horizontal angle (*φ*) is the angle measured anticlockwise from the positive y-axis to point projected in the X-Y-plane with −*π* ≤ *φ* ≤ *π*

Angles are considered spherical data, so they are analysed as a point (vector) on a unit sphere [39].

By examining a sample of n spherical data (*n*_1_, *n*_2_,…, *n*_n_), or (*θ*_1_, *φ*_1_)…(*θ*_n_, *φ*_n_) (polar coordinates) with corresponding direction cosines, the resultant length of the data is R:

(5) $$R=\sqrt{{\left(\sum x_{i}\right)}^{2}+{\left(\sum y_{i}\right)}^{2}+{\left(\sum z_{i}\right)}^{2}}$$

It can be observed that the following are the basic circular statistics for angles:

▪ Mean directions (*θ̅*, *φ̅*): Calculation of vectorial addition of n spherical data gives a vector resultant (*R*). The directions of these vectors are the mean directions. These angles are the average direction of each angular component.
▪ Mean module (*R̅*): The mean module can be obtained from the length of the vector resultant by:
  (6) $$\bar{R}=\frac{R}{n}$$

Because we work with the unit vectors, *R̅* is observed in the range (0, 1). Hence, if *R̅* = 1, then it signifies that all spherical data are coincident. However, *R̅* = 0 does not imply a uniform distribution.

▪ Concentration parameter (*κ*): This parameter is a measure of the concentration of data in a preferred orientation or distribution. If *κ* = 0, the distribution is uniform, but if κ tends to ∞ the distribution will be concentrated at one point. The Fisher distribution on spherical data (3D) is equivalent to von Mises distribution on circular data (2D), and also equivalent to a normal distribution in linear data (1D).
  Calculation the estimation of *κ*:
  when *R̅* = R/n ≥ 0.95
  *κ* = (n − 1)/(n − R)
  To n ≤ 16
  (7) $$\kappa ={\left(1-\frac{1}{n}\right)}^{2}.\frac{n}{n-R}$$
▪ Circular standard deviation (*ν*): This parameter for spherical data is similar to the S parameter for circular and linear data.

For more details [39].

### 2.3. Analysis of Uniformity and Distribution Error Data

Similar to the analysis of linear and circular data, while analysing spherical data, one should look for uniformity and normality (Fisher distribution) of distributions. The Fisher distribution is a symmetric unimodal distribution and can be considered as an analogue of the von Mises distribution in circular data; and normal distribution in linear data.

For spherical data, two tests are used:

▪ Rayleigh test: It is a uniformity test that detects a single modal direction in a sample of data. This test, developed by Lord Rayleigh in 1919, tests for uniformity against a unimodale alternate model, as assumed for the Fisher distribution. For n < 10, it compares the magnitude of the resultant vector, R, to a critical value. For n > 9, the test statistic, (3R)^2^/n, is tested with the chi-squared distribution test with three degrees of freedom at the 95% confidence level. La hypothesis of uniformity is rejected if this value is too large.
▪ Beran/Giné test: In 1968, R. J. Beran devised a statistic, based on the angle between pairs of sample directions, for testing uniformity against alternate models that are not symmetric with respect to the centre of the sphere [40]. E.M. Giné, in 1975, extended Beran’s work to the case where the data may be centro-symmetric [41]. The combined statistic, used for polar data, tests against both of these possibilities, by comparing the summed statistics to a critical value at the 95% confidence level.

## 3. A Study Case

### 3.1. Experiment Description

**Figure 2.**
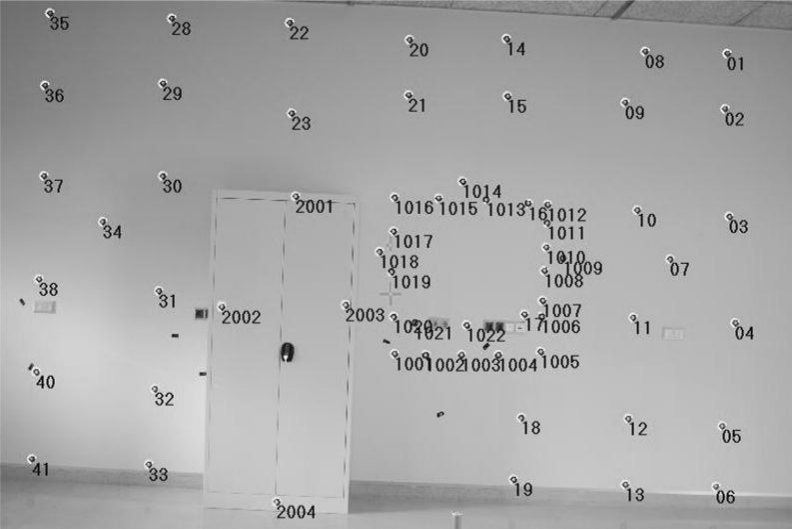
The wall with the distribution of targets used in the case study.

In this study case, the experiment was performed over a set of 53 targets or check points (CP) distributed over a wall of 9.80 × 3.22 m as shown in Figure 2. The targets were circular shaped, retroreflective material, with a centred cross. The center of the targets was measured with TLS, 3,000 to 400 points per target, and other equipment of higher accuracy (Proliner).

### 3.2. Materials

The equipment used in this work is listed below:

▪ TLS: a 3D long range TLS (TOF) Riegl LMS-Z390i, technical specification are provided in [42]. This equipment measures distances in a range of 1.5 to 400 meters, with a nominal precision of ±6 mm at 50 m distance in normal illumination and reflectivity conditions. The vertical field of vision has amplitude of 80 degrees and 360 degrees in the horizontal plane. It has a minimum angular resolution of 0.2 degrees and a maximum of 0.002 degrees, and the rate of measurement of points oscillates between 8,000 and 11,000 points per second. This scanner is used in combination with a calibrated Nikon D200 camera incorporating a CCD sensor, DX format (10.2 megapixels in total).
▪ The Proliner 5.7 system is especially designed for accurate measurement, gathered by locating a contact device, similar to a pen, on each point that defines the contours of the boat deck. This pen has a spherical tip that is joined to the machine by a cable. The 3D coordinates (X, Y, Z) of each point are stored in a memory device. As a result, all of the data can be exported to an ASCII file, where the 3D coordinates of all the points are included. Proliner is a machine that can be located in different positions: horizontal, vertical and tilted. The Proliner has a cable with a length of 5 meters, so the maximum distance that can be measured with the Proliner from a station position is 10 meters (in the absence of obstacles). Its precision in point coordinates, according to the manufacturer, is 0.3 mm.

Software package: All operations were performed using a variety of software such as RiSCAN PRO Software, Riegl^©^ used for the recording and alignment of clouds of points. The calculation of the parameters that link the two reference systems of measurement equipment used (Proliner to Riegl LMS) was performed with Matlab 7.1. The specific Statistical calculations were made with the following software packages:

▪ Statistical Package for the Social Sciences (SPSS): is a statistical analysis program, used for modular statistical analysis.
▪ Spheristat v2.2: is a specific software for spherical statistics for angular statistical analysis.
▪ VecStatGraph2D and VecStatGraph3D: are two packages in the R programming language (http://www.r-project.org), which is a language and environment for statistical computing and graphics and in addition available as Free Software under the terms of the Free Software Foundation's GNU General Public License in source code form. These two packages perform a 2D and 3D statistical analysis, both numerical and graphic, of a set of vectors and were developed for the Spherical graphics analysis proposed in this paper (http://gim.unex.es/VecStatGraphs2D, http://gim.unex.es/VecStatGraphs3D, respectively).

### 3.3. Data Collection, Preprocessing and Calculating Errors

**Figure 3.**
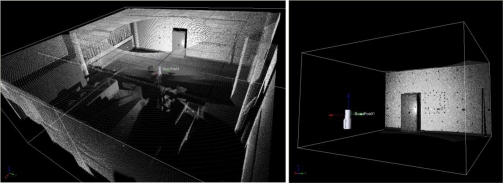
The room with the targets in the case study.

The whole room was scanned with an angular resolution of 0.2 degrees. Detailed scans were performed for every target, 3,000 to 4,000 points per target. They were automatically detected on the 2D overview of the initial point cloud through a contrast algorithm that allowed discriminating the retroreflective material of targets from the intensity values of the others materials in the scene. Once detected and scanned, circles are approximated, and corresponding centers are estimated with 0.001 standard deviation. Then, a detailed scan of the scene is performed at a 0.02-degree resolution (see Figure 3). Furthermore, the centre of each of the targets was also measured with Proliner through one station.

Once the measurements were made with both equipments, data were needed to be aligned (or unified) in a common reference system in order to calculate the differences of coordinates obtained at each check point, and thus the error was obtained at each point.

The alignment between the reference systems, Proliner and TLS, was performed using a rigid body transformation, more specifically, through 3D translation and three rotations in space. In this case, the translation was the origin point of reference of Proliner to the origin point of reference of TLS. The three cardanic rotations were made to coincide with the axes of both systems: *ω* in the X axis, *φ* in the Y axis and К on the Z axis.

Therefore, the unification of the two coordinate systems requires knowledge of some parameters. To calculate the transformation parameters five points (1, 6, 35, 41, and 1,025) were measured by measuring both teams. The distribution of these points was selected as the most optimal (see Figure 2). The results of the parameters used for the alignment of coordinates are shown in Table 1.

Error is defined as the difference between a measure and the correct value. In this study case, the error in each point is the difference between the location of each CP-TLS and its “true” coordinate measured by Proliner technology. This error was calculated for each CP. Therefore, a 3D error vector was calculated for each point of each CP. Furthermore, each vector was defined in terms of its modulus and angles.

### 3.4. Vectorial Error Analysis

The error analysis proposed of this paper was made in three parts. In the first part, the modular error component was calculated. This first part is similar to the conventional method based in the calculations of linear statistics. The second part was the directional error component analysis based in the calculations of spherical statistics. The third part was the most innovative part of the proposed analysis, which was developed by 2D and 3D graphics with two packages of the R programming language. In the last part, a study of the uniformity and normality of data distribution with errors data was achieved to complete data analysis.

#### 3.4.1. Modular Error Analysis

Modular accuracy evaluation of TLS was calculated for a set of 53 CPs. The basic statistics of the modulus was calculated. The results of the modular error components are summarized in Table 2. The mean, minimum, maximum, standard deviation and root mean square error (RMSE) values along the X, Y and Z axis and denoted Δx, Δy and Δz, respectively were calculated. The Δr vector is equal to the square root of [Δx^2^ + Δy^2^ + Δz^2^] at each point, and Δr is the modular or radial statistic; however, the Δr basic modular statistics (mean, minimum, maximum, standard deviation and RMS) is not equal to the square root of [(statistic Δx)^2^ + (statistic Δy)^2^ + (statistic Δz)^2^].

We can observe, in Table 2, that the basic statistics of the Δr modular error were 9.53 mm of mean error, 2.02 mm of minimum error, 18.39 mm of maximum error, 3.23 mm of RMSE and ±0.44 mm of standard deviation. We can observe that the modular error result is reasonable according to the technical characteristics of the TLS. This is one of the main advantages of the modular error statistical analysis.

#### 3.4.2. Angular Error Analysis

**Figure 4.**
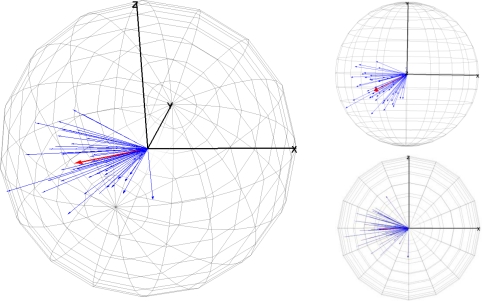
3D spherical graphic of errors with all vectors in blue and the mean vector in red.

**Figure 5.**
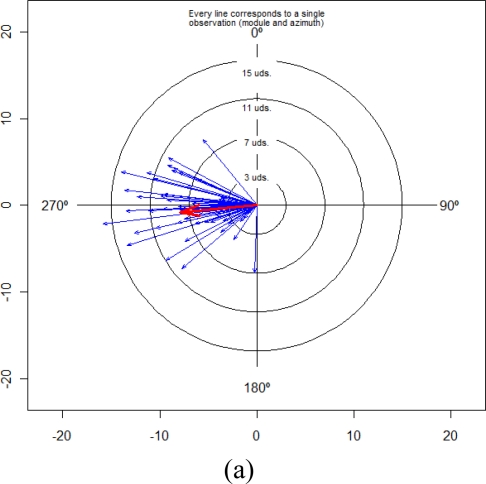

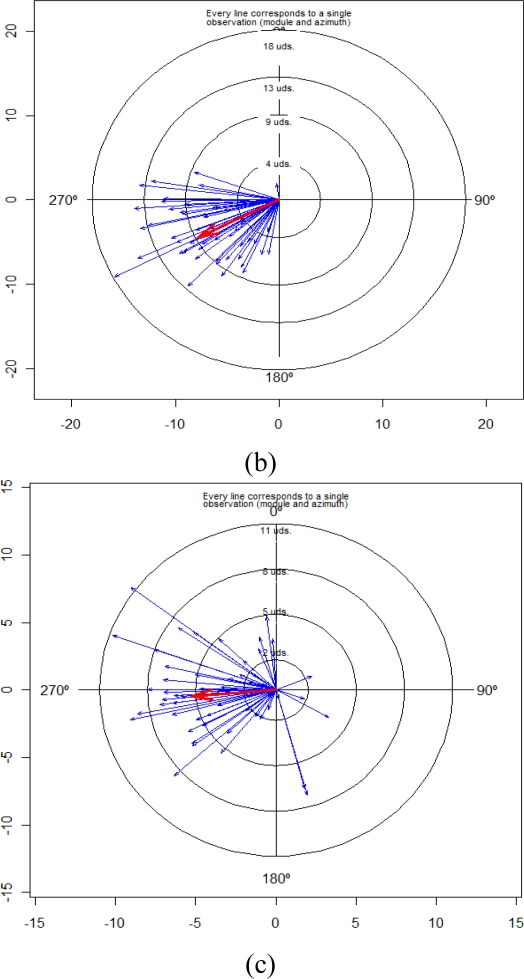
Data in circular diagram (2D). **(a)** XZ wall-plane; **(b)** XY ground-plane; **(c)** plane perpendicular to these planes.

**Figure 6.**
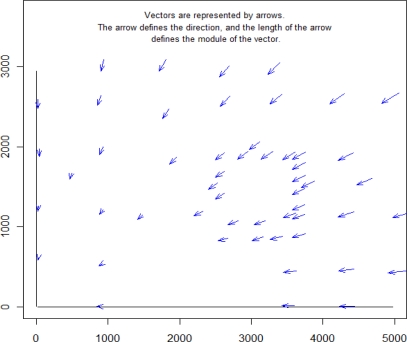
Map of vectors in the ZX wall-plane, 2D circular graphics analysis for each point in the wall (made with VecSartGraph2D).

The next approach in the angular accuracy evaluation of error TLS was calculated for a set of CPs using spherical statistics. In this part of the study, specific software—Spheristat V2.2—for spherical statistics was used to calculate the spherical statistical parameters. Spheristat is a commercially available program that offers basic functionality for the analysis of spherical data. On the other hand, the graphic part was made by two packages implemented in the R programming language, which was developed for this work. The results of the angular (vertical and horizontal) error components are summarized in Table 3 and Figures 4, 5 and 6.

The spherical statistics for angular error are mean direction directions (*θ̅*, *φ̅*), circular standard deviation (*ν*), mean module (*R̅*) of the all error vectors and the concentration parameter (*κ*). These statistics were explained in Section 2.2. The mean direction values were 249.7° of vertical angle and −3.8° of horizontal angle. The circular standard deviation value (*ν*) was 27.9°. On the other hand, a relatively high value of the mean module (*R̅*) 0.8, shows that they were not in a uniform distribution. As a last basic parameter for directions, the parameter (*κ*) is a measure of the concentration of data in a preferred orientation. The concentration parameter (*κ*) is 6.7. Therefore, if we consider that the value is not small, the data some show symmetrical distribution in relation to a preferred direction (see Figure 4).

#### 3.4.3. Graphical Error Analysis

Although angular error components are summarized in Table 3, the angular analysis is not complete without Figures 4, 5 and 6, which depict some of these parameters.

Figure 4 shows some three-dimensional representations of a sphere containing all error vectors. The error vectors radiate from the centre of the sphere and are represented by blue arrows. The mean vector was represented by the red arrow. This sphere allows a global view and a comparison of the modules and orientations of all vectors. Therefore, these 3D graphics are a complement to the data presented in Table 2 and Table 3 by providing a better and more complete analysis of the data. The 3D graphics of the Figure 4 were made with VecStatGraphs3D, a package in the R programming language.

Figure 5 shows two-dimensional representations of results of the projections of the previous sphere, shown in Figure 4, for the three main planes: XZ wall-plane (Figure 5(a)), XY ground-plane (Figure 5(b)), and the plane perpendicular to these planes (Figure 5(c)). The 2D graphics of the Figure 5 were made with VecStatGraphs2D, another package in the R programming language, which was developed for circular data. Finally, as the last graphic analysis proposed, Figure 6 shows a two-dimensional graphic that represent all of the errors in the XZ wall-plane, as in Figure 5(a), but with each error vector with the corresponding correct position, also achieved with the VecStatGraphs2D software package. In this study case, although the error modules are small, we can see a trend in the angular errors (see Figures 4 and 5). On the other hand, the modular error values at the top and right of the wall have higher values than at the bottom and left of the wall (see Figure 6).

#### 3.4.4. Uniformity and Distribution Error Analysis

The last step is to analyse the distribution of errors. Several tests were used to examine the uniformity and error distribution. For this part of the study, Spheristat software was used as well.

SpheriStat also tests whether the sample is from a uniform distribution using Rayleigh’s test of the magnitude of the resultant vector. This test compares the resultant vector, R, to a critical value at the 5% significance level. When R exceeds the critical value, the distribution cannot be considered to be uniform. In that case, SpheriStat compares the distribution to the Von Mises distribution, a circular equivalent to the Gaussian distribution. The result of Rayleigh’s test is that the distribution has a preferred trend, with an R value of 88.9% and an R critical value (5% level) of 23.8%.

The concentration parameter, *κ*, measures the spread of the distribution (a lower *κ* for a wider spread), and the 95% confidence angle, derived from the standard error of the mean, gives the uncertainty in the resultant direction. The concentration parameter (*κ*) of Von Mises model is 6.7. Therefore, if we consider that the value is not small, we can consider that the data show certain concentration.

The last step is to analyse the distribution of errors by applying several tests. The Rayleigh test is a uniformity test that detects a single modal direction in a sample of data. The result of the test of sample uniformity, Rayleigh’s test, is with a Rayleigh statistic of 115.97 better to a critical value of 7.81, so uniformity is rejected at the 95% confidence interval.

The result of the test of sample uniformity, Beran/Giné test, is with a Beran/Giné statistic of 37.24 better to a critical value of 2.75, so uniformity is rejected too at the 95% confidence interval.

## 4. Discussion and Conclusions

An improved methodology for the analysis of the vectorial errors of TLS data was presented in this paper. Although the method was applied to TLS data, it can also be applied to any three-dimensional data. In this study, we argue that the real nature of the positional error is vectorial, and thus error vectors should be analysed. These error vectors have three metric elements (one module and two angles), and these magnitudes were used for a complete analysis of the positional error.

In the case study presented as an example, 53 CP were measured by TLS with equipment to analyse and other equipment of higher accuracy (Proliner). We must take into account that the accuracy in the calculation of the errors was limited by the accuracy of the equipment used for this purpose. In the case study presented, the Proliner has ±0.3 mm accuracy compared to ±6 mm for TLS. Therefore, the calculation of errors was limited to ±0.3 mm of accuracy.

We might highlight that this case is not designed to find the sources of errors in the TLS instruments; instead, it is intended to show the benefits of spherical graphics and statistical analysis.

Results showed that the RMS modular error was 3.23 mm with ±0.44 mm of standard deviation. These values are reasonable if we know the technique characteristics of TLS. The first part of the analysis was performed as conventional analysis of the TLS error. The conventional analysis module provides information on the amount of error but not on the direction of error. This is analysed by means of the statistical analysis of the angles (Section 3.4.2). Figures 4, 5 and 6 show an important aspect of the error distribution that cannot be observed by analysing only the linear statistics: spatial error is clearly anisotropic. Error vectors from each CP show that TLS data are displaced to the east, but this displacement is not homogeneous because modular error values at the top and right of the wall have higher values than the bottom and left ones (Figure 6). The results show a preferential direction to the lower left corner.

In the angular error analysis the results convey the angular parameters (mean directions, circular standard deviation and concentration) but on a global form (Table 3). In the graphical error analysis (Section 3.5.3) both parts (modular and angular) are performed together (Figures 4, 5 and 6). These graphs show the angular trend mentioned above.

We think that this trend could be due to the position of the measuring equipment Prolainer, which was placed on the ground at the bottom left (point 0,0,0 of Prolainer). In Figure 6, if we move away from this point we can see that the error modules increase. This may be due to the great influence of the accuracy on the coordinates measured of each point which depends on the distance to the equipment. Therefore, we believe that Prolainer equipment has limited use to analyse the accuracy of a TLS scanner because: (a) the accuracy is quite limited to calculate the standard deviation of error; (b) accuracy is highly dependent on distance.

On the other hand, these results show the advantages of graphical error analysis using spherical statistics, which may reveal results that with conventional statistical analysis would be hidden.

In the uniformity and distribution error analysis (Section 3.4.4.), the study case showed that in this dataset errors (study case with Riegl LMS z390i) are not spatially uniform but not necessarily for all TLS data. These local effects are interesting and can be detected with the proposed methodology; otherwise, they may be unnoticed if the error analysis is restricted to linear statistics and/or a limited set of checkpoints. Spherical statistics permit the analysis of a set of spatial properties—the angular error component, “normality” or uniformity of vector distribution, and error isotropy and homogeneity—which cannot be taken into account in the error analysis based on traditional linear statistics, such as RMSE or standard deviation.

These graphics complete the analysis of data error with a joint view of the sphere of errors from several perspectives (3D view and their respective projections in the three principal planes), composing a sufficient set of charts to analyse the results. Finally, one of the purposes of this paper, besides presenting the potential offered by these graphs, was the advantage of its development in R for the scientific community, which is available as Free Software under the terms of the Free Software Foundation's GNU General Public License in source code form (http://www.r-project.org/). In our future work, we propose to focus on the uncertainty of the measurements of TLS in terms of repeatability.

## Figures and Tables

**Table 1.** Parameters transformation used to align reference systems (Proliner to TLS).

**Dx (mm)**	**Dy (mm)**	**Dz (mm)**	**Omega (*ω*)**	**Phi (*φ*)**	**Kappa (*κ*)**
9,057	1,703	1,055	−1°, 5438	−0°, 0133	−1°, 5879

**Table 2.** Basic statistic results for modulus analysis of errors.

**Basic-statistics**	**Δx error [mm]**	**Δy error [mm]**	**Δz error [mm]**	**Δr error [mm]**
Mean	−7.6	−3.83	−0.26	9.53
Min	−0.25	0.16	0.04	2.02
Max	−10.84	−10.18	−7.76	18.39
RMS	3.75	3.24	3.08	3.23
Standard deviation	0.51	0.44	0.42	0.44

Error analysis was calculated with a sample size 53 ICP.

Δx: X axis error; Δy: Y axis error; Δz: Z axis error; Δr: Radial error.

**Table 3.** Spherical statistics results for angular error analysis.

**Spherical-statistics**	
Mean directions (*θ̅*, *φ̅*)	239.7°/−3.8°
Circular Standard deviation (*ν*)	±27.9°
Mean module (*R̅*)	0.8
Concentration parameter (*κ*)	6.7

Calculated with Spheristat 2.2.
